# Trust of patients and families in mental healthcare providers and institutions: A cross-cultural study in Chennai, India and Montreal, Canada

**DOI:** 10.21203/rs.3.rs-2584056/v1

**Published:** 2023-02-17

**Authors:** Salome M. Xavier, Ashok Malla, Greeshma Mohan, Sally Mustafa, Ramachandran Padmavati, Thara Rangaswamy, Ridha Joober, Norbert Schmitz, Howard C. Margolese, Srividya N. Iyer

**Affiliations:** McGill University; Douglas Mental Health University Institute; Schizophrenia Research Foundation (SCARF); Douglas Mental Health University Institute; Schizophrenia Research Foundation (SCARF); Schizophrenia Research Foundation (SCARF); Douglas Mental Health University Institute; Douglas Mental Health University Institute; McGill University Health Centre; Douglas Mental Health University Institute

**Keywords:** Trust, Early Intervention Services, Psychosis, Cross-cultural

## Abstract

**Purpose:**

Cross-cultural psychosis research has typically focused on a limited number of outcomes (generally symptom-related). It is unknown if the purported superior outcomes for psychosis in some low- and middle-income countries extend to fundamental treatment processes like trust. Addressing this gap, we studied two similar first-episode psychosis programs in Montreal, Canada and Chennai, India. We hypothesized higher trust in healthcare institutions and providers among patients and families in Chennai at baseline and over follow-up.

**Methods:**

Upon treatment entry and at months 3, 12 and 24, trust in healthcare providers was measured using the Wake Forest Trust scale and trust in the healthcare and mental healthcare systems using two single items. Non-parametric tests were performed to compare trust levels across sites and mixed-effects linear regression models to investigate predictors of trust in healthcare providers.

**Results:**

The study included 333 patients (Montreal=165, Chennai=168) and 324 family members (Montreal=128, Chennai=168). Across all timepoints, Chennai patients and families had higher trust in healthcare providers and the healthcare and mental healthcare systems. The effect of site on trust in healthcare providers was significant after controlling for sociodemographic characteristics known to impact trust. Patients’ trust in doctors increased over follow-up.

**Conclusion:**

This study uniquely focuses on trust as an outcome in psychosis, via a comparative longitudinal analysis of different trust dimensions and predictors, across two geographical settings. The consistent differences in trust levels between sites may be attributable to local cultural values and institutional structures and processes and underpin cross-cultural variations in treatment engagement and outcomes.

## 1. Introduction

In the context of healthcare, trust has been understood as stemming from the acceptance of the uncertainty and vulnerability that arises from the illness experience and the belief that one’s best interests (as a patient) will be taken care of [1, 2]. It has been conceptualized as a multidimensional construct, incorporating aspects such as fidelity (to the patients’ interests above all); competence (both technical and communication-related); honesty; confidentiality; and global trust (an overarching trust component that encompasses more subtle aspects of interpersonal communication) [3]. Components of patients’ trust usually pertain to specific healthcare professionals, but they may also apply to the medical profession as a whole or to healthcare institutions. Interpersonal and institutional dimensions of trust have been reported to be inter-related, despite being considered as different constructs [3–5].

Overall, trust is known to be an essential ingredient in therapeutic relationships and considered the foundation of patient-centered care [6]. It is also a major focus of medical ethics [7, 8]. Trust in healthcare institutions and healthcare providers have been theorized and found to impact self-reported health, help-seeking, engagement with services and treatment compliance [9–14].

Previous studies have reported conflicting and at times inconclusive results regarding the association between patients’ sociodemographic characteristics and their trust in healthcare institutions and providers [2, 15]. When differences between sociodemographic groups were reported, they seemed to lean towards lower trust levels among men, younger and more educated individuals [16–21]. Continuity of care and flexibility to adapt services to patients’ preferences have been reported to enhance trust in different settings [22, 23]. Furthermore, issues of communication, partnership and power balance have also been described as impactful for building and preserving trust [24]. Importantly, the way in which these aspects matter to individuals and communities is influenced by previous experiences, conceptualizations and expectations about healthcare systems and about the nature of patient-provider relationships [25]. These experiences and expectations are in turn largely determined by social and cultural factors and may become particularly salient when comparing different countries [26, 27]. Despite the interest in and relevance of exploring and comparing trust in different healthcare settings worldwide, previous studies have paid little attention to trust within healthcare institutions from the Global South. A few extant published studies suggest differences in trust levels and valued aspects of trust between countries [28–32].

Considering the long history of fear and stigma associated with psychiatric institutions and the specificities of mental health disorders and their treatment, trust building is especially important – and may be particularly challenging - in the context of mental healthcare [21, 33, 34]. Non-coerciveness, safety, dignity, caring, concern, confidentiality and continuity of care are of distinctive relevance in mental healthcare [35–37]. Trust building may be particularly demanding in the provision of care for patients with psychosis [38]. Early intervention for psychosis (EIS), which is the most advocated model of care for first-episode psychosis [39], emphasizes a patient-centred and recovery-focused approach. These services also place great focus on continuity of care, with patients receiving services throughout their tenure in the program from the same core team (typically, a physician and case manager). However, its simultaneous focus on risk management and medication adherence has been reported to hinder dynamics of trust between patients and providers [40–42].

The present study is part of a larger cross-cultural research project [43, 44] conducted between 2012 and 2018 in similarly structured EIS, in Montreal (Canada) and Chennai (India). Building from previous (and much disputed) findings suggesting that psychosis outcomes could be better in economically developing (vs developed) countries [45], the larger study aimed to compare these sites on multiple clinical, subjective and functional outcomes. It also aimed to study core processes (such as service engagement, family involvement and trust) that may be shaped by sociocultural contexts but have rarely been examined in prior cross-cultural psychosis research. These processes are worthy of examination not only because they may underpin inter-site differences in outcomes but also because they are increasingly recognized as valuable in their own right [7, 46, 47].

In this study, our focus was on differences in the level of trust in healthcare providers among patients and families in Chennai and Montreal. This line of enquiry was sparked by the finding from pilot phase focus groups that patients and family members in Chennai trusted their providers and program more than their Montreal counterparts did [48]. Since family involvement is critical for patients’ access to services, engagement and recovery [12, 43, 44, 49], we also deemed it important to assess trust among family members. Likewise, because early intervention services are provided by multidisciplinary teams offering case management and psychiatric follow-up [50], the care providers in whom we measured trust included not only doctors but also case managers.

## 2. Aims And Hypotheses

Our primary aim was to compare the trust levels of patients and their family members receiving care at two early intervention services in Chennai and Montreal.

At baseline, we predicted that trust in the medical profession (without reference to any specific doctor), and in the healthcare and mental healthcare systems (i.e., institutional trust prior to their experience with EIS) would be higher among Chennai patients and families compared to their Montreal counterparts, after accounting for sociodemographic factors associated with trust levels (such as age, gender or educational level) and for institutional trust levels.

At follow-up, we predicted that patients’ and families’ trust in their doctors and case managers and in the mental healthcare system (now after experiencing care in EIS) would be higher in Chennai than in Montreal, after accounting for sociodemographic factors associated with trust levels (such as age, gender or educational level) and for baseline trust levels.

As exploratory analyses, considering that continuity of care has been previously associated with higher trust levels [35–37], we assessed whether trust in doctors and case managers changed over time (from Month 3 to Month 24 of follow-up) at both sites. Finally, acknowledging the relevance of family involvement in the context of early intervention [12, 43, 44, 49], we compared trust between family members and patients.

## 3. Methods

### 3.1. Setting and sample

This study was conducted in two McGill University-affiliated early intervention services in Montreal, Canada (Prevention and Early Intervention Program for Psychosis/PEPP), and one early intervention service in Chennai, India (Schizophrenia Research Foundation/SCARF). PEPP is a publicly funded program that is part of the healthcare system and SCARF is a non-governmental non-profit organization and a World Health Organization (WHO) collaborating center. Both programs follow international guidelines for early intervention services [51–53], work on an open-referral basis and offer services that focus on early, assertive, phase-specific and recovery-oriented care [54, 55]. At both programs, patients are followed for two years by a multidisciplinary team and receive a wide range of psychosocial and medical services including assertive case management, family psychoeducation, flexible use of antipsychotic medication and, as needed, other individual and family psychosocial interventions. Although both programs share many commonalities, some adaptations were made to the Indian context, including a more flexible access to services (e.g., services provided without an appointment or by phone), the implementation of home-based interventions such as cognitive training focused on household chores, and a particular emphasis on family involvement [55].

#### 3.1.1. Inclusion criteria

- Primary diagnosis of a schizophrenia-spectrum or affective psychotic disorder (DSM-IV classification)
- Age between 16–35 years old
- Ability to communicate in Tamil or English (Chennai) and in French or English (Montreal).

#### 3.1.2. Exclusion criteria

- Treatment with antipsychotic medication for more than one month
- Primary diagnosis of a pervasive developmental disorder, organic psychosis or substance abuse/dependence. Patients with concurrent diagnoses of substance abuse/dependence were not excluded.
- IQ below 70

Family members recruited for this study included parents, legal guardians, grandparents, siblings, spouses/partners, extended family members, friends and roommates.

### 3.2. Measures

Assessments were administered by staff who went through similar training, using measures that had been previously administered at both study sites [48]. Several measures were taken to ensure quality of assessments, including inter-rater reliability sessions and centralized data management and verification. Patients’ and family members’ sociodemographic data were assessed at baseline through a questionnaire developed specifically for the larger study.

Interpersonal trust levels were measured using the Wake Forest Trust in Medical Profession Scale and the Wake Forest Physician Trust Scale [2, 56] (suppl. table 1). Both are short 5-item forms, assessing self-reported trust in the medical profession or towards a specific physician, respectively. For the purpose of this study, the Wake Forest Physician Trust Scale was also modified to assess trust in case managers and to be administered to family members. Although the Wake Forest Trust scales have not been extensively used in the context of mental healthcare [57], they were chosen for their demonstrated adequate psychometric properties, brevity and simplicity [56]. Permission for adaptation and translation was sought from the lead developers of these instruments. All scales were translated from English into Tamil and French and back-translated using standardized procedures recommended for cross-cultural research [58–60]. Each item of the scales is scored from 1 (strongly disagree) to 7 (strongly agree), with higher scores indicating higher trust.

Institutional trust was measured with two self-rated items, one to assess trust in the healthcare system (How much do you trust the health care system?) and the other to assess trust in the mental healthcare systems (How much do you trust the mental health care system?). While the former has been applied in previous studies [14, 61], the latter was created for this study. These questions are scored from 1 (no trust at all) to 5 (very high trust).

We evaluated the psychometric properties of the modified Wake Forest scales and the single self-rated item on trust in the mental healthcare system. Overall, we found that reliability and internal consistency estimates were adequate for patients and families, in Montreal and Chennai (suppl. table 2).

Patients and family members completed the trust measures at different time points. At baseline (entry into the early intervention service), the self-reported trust measures included the Wake Forest Trust in Medical Profession Scale and the two additional questions on institutional trust (specifically, trust in the healthcare system and in the mental healthcare system). During follow-up (at months 3, 12 and 24 after baseline), the Wake Forest Physician Trust Scale, its adapted version for case managers and the single-item questionnaire on trust in the mental healthcare system were distributed.

### 3.3. Data analysis

As different trust measures were administered at baseline and during follow up, data analysis was performed both cross-sectionally and longitudinally. Descriptive data were presented using proportions for categorical variables and means, standard deviations, medians and IQRs (interquartile ranges) for continuous variables. Total scores from 5-item trust scales were standardized to a scale from 1 to 5 to facilitate comparisons. Chi-square statistics were performed to examine differences between sites regarding categorical variables and t-tests were computed to assess site differences for continuous and normally distributed sociodemographic variables. To assess whether continuous sociodemographic variables were associated with trust measures, Spearman correlations were used, due to the right skewness of the trust data. In order to analyse differences in trust levels between sites and stakeholder groups, non-parametric Mann-Whitney tests were performed. Effect sizes were calculated using eta squared (ηp^2^) coefficients, classified as small: (η^2^ = 0.01), medium (η^2^ = 0.06) and large (η^2^ = 0.14) [62].

Linear regression models were fitted to assess predictors of trust in the medical profession at baseline and linear mixed effects models (autoregressive covariance structure) were fitted to assess predictors of trust in doctors and trust in case managers during follow-up (months 3, 12 and 24). Independent variables were chosen based on study hypotheses. The choice of models was based on theoretical assumptions and likelihood criteria (Bayesian Information Criterion). Statistical analyses were executed using SPSS 21.0 for Windows.

## 4. Results

### 4.1. Descriptive statistics

#### 4.1.1. Socio-demographics

The total baseline sample included 333 patients with first-episode psychosis from Montreal (N = 165) and Chennai (N = 168), and 324 family members from Montreal (N=128) and Chennai (N=168). At baseline, at both sites, most patients were in their mid-twenties, had completed high school level education and lived with their families. Participants from Montreal were significantly younger, more often male and single. Most family members belonged to the age group between 50–60 years old, were female, patients’ parents, and had high school (or higher) education. Family members from Chennai were more likely to be female, to have less than high school education and to be older than Montreal family members (table 1).

#### 4.1.2. Respondents versus non-respondents

At Baseline, 82/165 patients from Montreal and 4/168 patients from Chennai did not complete the trust scales. Within the family members’ group, 61/128 participants from Montreal and 4/168 participants from Chennai did not complete the trust scales at baseline. During follow-up, 36/165 patients and 21/128 family members from Montreal; and 3/168 patients and 33/168 family members from Chennai did not respond to both the scale on trust in doctors and the scale on trust in case-managers at any of the follow-up times. Overall, there were no significant differences between respondents and non-respondents on any of the assessed sociodemographic variables (baseline and follow-up) and in terms of previous assessments of trust (follow-up) except for family members in Chennai, among whom follow-up respondents had reported higher trust in the mental healthcare system at baseline (Mann-Whitney U = 2437; p = 0.041; η^2^ = 0.015).

#### 4.1.3. Trust scales

At both sites, at both baseline and follow-up, among patients and families, the trust scales’ distribution of responses was right skewed, with medians for interpersonal trust (trust in the medical profession at baseline and in doctors and case managers at follow-up), and institutional trust (trust in healthcare and mental healthcare institutions at baseline and follow-up) ranging between 4 and 5. An exception to this was patients’ trust in the medical profession at baseline, with a lower median at 3.8 (suppl. tables 3 and 4). At baseline, at both sites, correlations between trust measurements were strong for both stakeholder groups. At each follow-up time point, trust levels in doctors and in case managers were strongly correlated and moderately correlated with trust in the mental healthcare system [63] (suppl. tables 5–7).

### 4.2. Comparison of trust levels between sites

At baseline, patients and family members in Chennai had higher trust in the medical profession, and in the healthcare and mental healthcare institutions, compared to participants in Montreal (table 2). Similarly, during follow-up, both patients and family members in Chennai had higher trust in their doctors, case managers, and in the mental healthcare system, compared to their counterparts in Montreal (table 3).

### 4.3. Comparison between stakeholders

At baseline, in Chennai, families had higher trust in the medical profession and in institutions (healthcare and the mental healthcare system) compared to patients. In Montreal, no statistically significant differences were found between patients’ and families’ trust levels in institutions and in the medical profession (table 4). During follow-up, differences between stakeholders were often statistically significant (across different timepoints) in Chennai, with families scoring higher than patients in all trust measures at month 12 and 24. In contrast, no significant differences were found between patients’ and family members’ levels of trust in Montreal, at months 3 and 12. When differences were found (at month 24), patients showed higher trust than family members in both doctors and the mental healthcare system (table 5).

### 4.4. Predictors of patients’ and family members’ trust in the medical profession [baseline] and in healthcare professionals (doctors and case managers) [follow-up]

#### 4.4.1. Baseline: Multiple linear regression (Outcome: Trust in the medical profession)

In the final model for patients (F (3, 223) = 58.73; p<0.001; adjusted R squared: 0.43) trust in the medical profession was significantly associated with site (Montreal as reference category) (β_1_ = 0.22; p < 0.001), trust in the healthcare system (β_1_ = 0.4; p<0.001) and trust in the mental healthcare system (β_1_ = 0.22; p<0.001). The effect of site (Chennai > Montreal) on trust in the medical profession remained significant when variables of institutional trust and sociodemographic variables (age, gender and educational level) were added to the model. In the final model for family members (F (3, 210) = 97,18; p<0,001; adjusted R squared: 0.58) trust in the medical profession was significantly associated with site (Montreal as reference category) (β_1_ = 0,29; p<0,001), trust in the healthcare system (β_1_ = 0,29; p<0,001) and trust in the mental healthcare system (β_1_ = 0,30; p<0,006). The effect of site (Chennai>Montreal) remained significant when the variables of institutional trust and sociodemographic variables (age, gender and educational level) were added to the model (suppl. table 8).

#### 4.4.2. Follow-up: Linear mixed-effects regression models (Outcome: Trust in doctors)

In the patients’ group, site (Chennai as the reference category) (β_1_ = − 0,38; p<0,001), baseline trust in the medical profession (β_1_ = 0,33; p<0,001) and time (β_1_ = 0,006; p=0,007) were found to be significant predictors of trust in doctors during follow-up. These effects remained significant even when other sociodemographic variables (age, gender and education) were added to the model. Among family members, site (Chennai as the reference category) (β_1_ = −0,45; p<0,001), baseline trust in the medical profession (β_1_ = 0,23; p<0,001) and age range (higher trust for older family members) were found to be significant predictors of trust in doctors. These effects remained significant even when other sociodemographic variables (gender and educational level) were added to the model. The effect of site (Chennai>Montreal) was sustained when baseline trust in the medical profession was added to the model. Trust in doctors did not change significantly over time, among family members, both in simple and multinomial models (suppl. table 9).

#### 4.4.3. Follow-up: Linear mixed-effects regression models (Outcome: Trust in case managers)

In the patient group, site (Chennai as the reference category) (β1 = −0,26; p<0,001) and baseline trust in the mental healthcare system (β1 = 0,10; p<0,001) were found to be significant predictors of trust in case managers during follow-up. These effects remained significant even when other sociodemographic variables were added to the model. Among family members, site (Chennai as the reference category) (β1 = −0,45; p<0,001), baseline trust in the medical profession (β1 = 0,23; p<0,001) and age range (higher trust for older individuals) were found to be significant predictors of trust in case-managers. These effects remained significant even when other sociodemographic variables and baseline trust in the medical profession were added to the model. Trust in case managers did not change significantly over time for both stakeholder groups (suppl. table 10).

## 5. Discussion

As hypothesized, compared to their counterparts in Montreal, both patients and family members from Chennai scored higher on all self-reported measures of institutional trust (in the healthcare and mental healthcare systems) and interpersonal trust (in the medical profession at baseline and in their own doctor and case manager during follow-up). The effect of site was still significant even when other variables (such as age, gender, educational level), known to influence trust, were added to the models.

Site differences in trust are likely to be influenced by the social, cultural and political contexts that underpin peoples’ realities at both sites (Montreal and Chennai). Sociocultural contexts also shape public opinion and determine previous experiences with healthcare services and providers by influencing the availability, access and quality of services received. All in all, contexts mould expectations regarding institutions and healthcare professionals, shaping the ways in which therapeutic relationships are conceptualized and enacted in the context of healthcare [25, 64, 65]. Previous studies have also identified differences in institutional [27] and interpersonal [26] trust between countries. Such differences have been attributed to distinct local cultural values (thought to favour or hinder trust as a general societal value), and to structural differences in healthcare systems in terms of availability, affordability and quality of services.

It has been suggested that trust may be higher in collectivistic (than in individualistic) societies, characterized by a higher sense of group and interpersonal cooperation, greater emphasis on hierarchy and authority, and less concern with avoiding uncertainty. Our study results align with this perspective, since Canada and India could arguably be placed at the opposite poles of the individualistic-collectivistic spectrum [66–68]. However, care should be taken when applying such classifications in a simplistic fashion, as cultural value orientations are not generalizable to whole populations and may differ substantially from individual-level and intra-society values. Moreover, in India, where cultural values seem to favour higher trust as a general value, it has been reported that trust towards others, the government and science is low, as compared to individualistic societies [69]. Likewise, accounts of low trust towards health institutions have been reported in India [70]. It may also be that the trust that participants in Chennai placed in the mental healthcare/healthcare systems and care providers was driven by the setting of this study being SCARF, which is an esteemed NGO valued by the local community for its free high-quality services [71]. Similar levels of high trust in other healthcare systems and providers may not be evident in the Indian populace. A similar argument could be made about PEPP, where relatively high trust levels were also reported. Both PEPP and SCARF’s early intervention services were built under a care model that was initiated to break with previous practices by facilitating access, promoting engagement and providing individualized and stage-specific treatment. However, such services might stand out more in a context like India where resources are limited and where a universal public healthcare system is not implemented [70, 72].

At baseline, trust in the medical profession was associated with trust in the healthcare and mental healthcare system, for both patients and family members. Age was a significant predictor of trust for only family members, with older individuals having higher trust levels, as previously reported in the literature [16–21], suggesting a generational effect that could be partly related with previous healthcare experiences. Patients’ and families’ trust in the medical profession at baseline predicted their trust in their doctors (and case managers) over the course of follow-up. This finding is aligned with previous studies reporting a positive correlation between public trust in doctors and trust in specific physicians [3].

When comparing stakeholder groups, results differed by site, with family members from Chennai reporting consistently higher trust levels in both institutions and healthcare providers. In Montreal, however, no differences were found between patients’ and families’ trust levels, at most timepoints. This is particularly relevant given that in other studies from this cross-cultural project, family involvement was found to be higher in Chennai than in Montreal, and valuable for both promoting engagement and better clinical outcomes [43, 44].

This study has several strengths, which include its prospective design and well-defined inclusion and exclusion criteria, the similarity of the care models and protocols at both sites, the rigorous staff training, centralized data management and verification, and the strong psychometric properties of the instruments used. Furthermore, it is unique and innovative in its focus on trust in early intervention services for psychosis, while assessing trust across countries in a nuanced way, considering different trust dimensions (interpersonal and institutional), from different points of view (i.e., from patient and family member perspectives). Several study limitations should also be noted. At both sites, all stakeholders reported high levels of institutional and interpersonal trust, with medians ranging between 3.4 and 5 (out of a total of 5 points), suggesting that responses could have been influenced to some extent by social desirability. However, both families and patients were asked to report their trust levels at an early stage of contact with services. Also, all trust scales showed good to excellent psychometric properties at both study sites. It should also be pointed out that not all chosen measures were developed specifically for this study and hence were not specifically adapted to the study populations who could conceptualize and evaluate trust differently. For instance, previous studies from South India have reported that, for doctors to be trusted, it is more important that patients experience respect, loyalty, comfort, personal involvement by the care provider, cultural competence and the assurance of being treated (regardless of the ability to pay), whereas the experience of individuality, shared responsibility and confidentiality tend to be emphasized in Western contexts [32, 73–75]. Furthermore, the validity of the institutional trust measures could be questioned in the Indian context, since evaluating systems may be a difficult task when there are no clear systems in place. Nonetheless, both the questions regarding institutional trust and the Wake Forest trust scales (and adaptations) are general and focused on aspects of trust that are relevant at both settings (e.g.: overall trustworthiness of care-provider/institution; subjective competence; careful and attentive behaviour by the care-provider). A considerably higher number of participants from Montreal did not complete the trust scales at baseline. Even though there were no differences (in terms of the assessed demographic variables) between responders and non-responders for both patients and family members, we cannot exclude the possibility that the non-responders would have reported lower (or higher) trust levels. If so, site differences may have been underestimated (or overestimated). Of note, differential response rates may be reflective of contextual differential engagement with services [43], which in turn may be underpinned by contextually and culturally shaped notions of trust [66]. Site differences are likely to reflect the impact of a very complex array of factors that we have only partially accounted for in this study. Trust is a two-way street and, in this study, we have not measured care provider attitudes that could differ between sites and that may hinder or facilitate trust. Finally, future studies examining trust in cross-cultural contexts would benefit from including additional variables known to influence trust and which may differ between sites. Some examples would be the assessment of care provider attitudes and institutions’ attributes, self-assessment of stigma and self-stigma (reported to negatively impact trust) [21] and of aspects of pathways to care that might impact trust (e.g.: police involvement, etc.) [76]. Further studies set to qualitatively explore the reasons and mechanisms underlying site differences in trust could be particularly enlightening and result in a more fine-grained understanding about what trust means, what contributes to it and what it implies for patients, family members and clinicians across contexts.

## Figures and Tables

**Table 1.** Sociodemographic variables and characteristics at entry for treatment for patients and family members*

PATIENTS				
Variable	Montreal	Chennai	Statistics	p**
Age at entry, years: mean (s.d.)	24.20 (5.3)	26.60 (5.24)	t (331) = 4.15	<0.001
Gender, n (%)			χ^2^(2) = 12.37	0.002
Men	110 (66.7)	82 (48.8)		
Women	54 (32.7)	86 (51.2)		
Transgender	1 (0.6)	0		
Total	165	168		
Education, years: mean (s.d.)	12.24 (2.63)	11.75 (3.9)	t (293.938) = 1.34	0.182
Education level, n (%)			χ^2^(1) = 0.03	0.868
Less than high school	44 (27.2)	47 (28)		
High school or more	118 (72.8)	121 (72)		
Total	162	168		
Occupational status, n (%)			χ^2^(3) = 30.0	<0.001
Student	40 (29.0)	24 (14.4)		
Paid employment	35 (25.3)	25 (15.0)		
Homemaker	7 (5.1)	40 (24.0)		
Unemployed	56 (40.6)	78 (46.7)		
Total	138	167		
Relationship status, n (%)			χ^2^(2) = 50.51	<0.001
Single	149 (90.9)	95 (56.5)		
Married or common law relationship	13 (7.9)	62 (36.9)		
Separated, divorced or widowed	2 (1.2)	11 (6.5)		
Total	164	168		
Living situation, n (%)			χ^2^(3) = 22.95	<0.001
Alone	16 (10.0)	2 (1.4)		
With family	125 (78.1)	140 (96.6)		
With friend or roommate	16 (10.0)	2 (1.4)		
In residence, group home or homeless	3 (1.9)	1 (0.7)		
Total	160	145		
FAMILY MEMBERS				
Variable	Montreal	Chennai	Statistics	p**
Age range, n (%)			χ^2^(5) = 25.28	<0.001
21–30	11 (8,9)	13 (8.0)		
31–40	41 (33.0)	24 (14.7)		
41–50	19 (15.3)	30 (18.4)		
51–60	52 (42.0)	74 (45.4)		
61–70	1 (0.8)	20 (12.3)		
71–80	0 (0.0)	2 (1.2)		
Total	124	163		
Gender, n (%)			χ^2^(1) = 20.15	<0.001
Men	66 (53)	46 (27)		
Women	58 (47)	122 (73)		
Total	124	168		
Education level, n (%)			χ^2^(1) = 5.99	<0.001
Less than high school	2 (1.6)	13 (8.2)		
High school or more	122 (98.4)	146 (91.8)		
Total	124	159		
Family member category			χ^2^(3) = 6.23	0.101
Parent	69 (55.6)	117 (69.6)		
Spouse/partner/boyfriend/girlfriend	30 (24.2)	29 (17.3)		
Sibling	17 (13.7)	16 (9.5)		
Other	8 (6.5)	6 (3.6)		
Total	124	168		

* The sample sizes vary because of missing data.

** Significance level set at < 0.05

**Table 2:** Comparison of trust measures between sites at baseline, for patients and family members

PATIENTS					
	Chennai - median (IQR)	Montreal-median (IQR)	Test Statistic* - U	Effect size (2)	p**
Trust healthcare system	4 (2)	4 (1)	5043	0.012	0.012
Trust mental healthcare system	4 (2)	4 (1)	4767	0.04; 0.40	<0.01
Trust in medical profession	3.9 (0.4)	3.5 (1.1)	4398	0.08; 0.60	<0.001
FAMILY MEMBERS					
	Chennai - median (IQR)	Montreal-median (IQR)	Test Statistic* - U	Effect size (2)	p**
Trust healthcare system	5 (1)	4 (1)	8185	0.26	<0.001
Trust mental healthcare system	5 (1)	4 (1)	7810	0.29	<0.001
Trust in medical profession	4.7 (0.7)	3.4 (0.9)	8891	0.36	<0.001

* Mann-Whitney Test;

** Correlation is significant at the 0.05 level (2-tailed).

**Table 3:** Comparison of trust measures between sites during follow-up, for patients and family members

PATIENTS					
*Month 3*					
	Chennai - median (IQR)	Montreal-median (IQR)	Test Statistic* - U	Effect size (2)	p**
Trust mental Healthcare system	5 (1)	4 (1)	2945	0.16	<0.0001
Trust in doctor	4.7 (0.8)	4.3 (1.1)	3531	0.14	<0.0001
Trust in case manager	4.6 (0.7)	4.2 (1.2)	3257	0.16	<0.0001
*Month 12*					
	Chennai - median (IQR)	Montreal-median (IQR)	Test Statistic* - U	Effect size (2)	p**
Trust mental healthcare system	5 (1)	4 (2)	3424	0.06	<0.001
Trust in doctor	4.6 (0.7)	4.2 (1.2)	3519	0.13	<0.001
Trust in case manager	4.7 (0.7)	4.2 (1.1)	4111	0.08	<0.001
*Month 24*					
	Chennai – median (IQR)	Montreal-median (IQR)	Test Statistic* - U	Effect size (2)	p**
Trust mental healthcare system	4 (1)	4 (2)	3053	0.02	<0.001
Trust in doctor	4.9 (0.5)	4.3 (1.1)	2613	0.09	<0.001
Trust in case manager	4.7 (0.7)	4.3 (0.9)	2891	0.05	<0.001
FAMILY MEMBERS					
*Month 3*					
	Chennai - median (IQR)	Montreal-median (IQR)	Test Statistic* - U	Effect size (2)	p**
Trust mental Healthcare system	5 (1)	4 (1)	1950	0.23	<0.001
Trust in doctor	5 (0.7)	4.1 (0.9)	2020	0.21	<0.001
Trust in case manager	5 (0.7)	4.1 (0.8)	2206	0.20	<0.001
*Month 12*										
	Chennai - median (IQR)	Montreal-median (IQR)	Test Statistic* - U	Effect size (2)	p**
Trust mental healthcare system	5 (0)	4 (1)	1260	0.39	<0.001
Trust in doctor	5 (0.5)	4.3 (80)	1905	0.48	<0.001
Trust in case manager	5 (0.5)	4.3 (81)	2048	0.26	<0.001
*Month 24*										
	Chennai - median (IQR)	Montreal-median (IQR)	Test Statistic* - U	Effect size (2)	p**
Trust mental healthcare system	5 (0)	3 (1)	930	0.35	<0.001
Trust in doctor	5 (0.4)	3.9 (1)	1037	0.32	<0.001
Trust in case manager	5 (0.4)	4.1 (1)	1771	0.19	<0.001

* Mann-Whitney Test;

** Correlation is significant at the 0.05 level (2-tailed)

**Table 4:** Comparison of trust measures between patients and families at baseline, in Montreal and Chennai

MONTREAL					
	Patients – median (IQR)	Family members-median (IQR)	Test Statistic* - U	Effect size (2)	p**
Trust healthcare system	4 (1)	4 (1)	2124	0.008	0.265
Trust mental healthcare system	4 (1)	4 (1)	2041	0.016	0.119
Trust in medical profession	3.5 (1.1)	3.4 (0.9)	2499	0	0.897
CHENNAI					
	Patients – median (IQR)	Family members - median (IQR)	Test Statistic* - U	Effect size (2)	p**
Trust healthcare system	4 (2)	5 (1)	8888	0.086	<0.001
Trust mental healthcare system	4 (2)	5 (1)	8717	0.09	<0.001
Trust in medical profession	4 (0.4)	4.7 (0.7)	4054	0.36	<0.001

* Mann-Whitney Test;

** Correlation is significant at the 0.05 level (2-tailed).

**Table 5:** Comparison of trust measures between patients and families during follow-up, in Montreal and Chennai

MONTREAL					
*Month 3*					
	Patients - median (N)	Family members-median (N)	Test Statistic* - U	Effect size (2)	p**
Trust mental healthcare system	4 (1)	4 (1)	2768	0,006	0.286
Trust doctors	4.3 (1.1)	4.1 (0.9)	3457	0.001	0.662
Trust case managers	4.2 (1.2)	4.1 (0.8)	3475	0.002	0.675
*Month 12*					
	Patients - median (IQR)	Family members-median (IQR)	Test Statistic* - U	Effect size (2)	p**
Trust mental healthcare system	4 (2)	4 (1)	2422	0.012	0.123
Trust doctors	4.2 (1.2)	4.3 (0.8)	3608	0	0.878
Trust case managers	4.2 (1.1)	4.3 (0.8)	3167	0.009	0.210
*month 24*					
	Patients - median (IQR)	Family members-median (IQR)	Test Statistic* - U	Effect size (2)	p**
Trust mental healthcare system	4 (2)	3 (1)	914	0.06	0.008
Trust doctors	4.3 (1.2)	3.9 (1)	1040	0.07	0.007
Trust case managers	4.3 (0.9)	4.1 (1)	1369	0.009	0.329
CHENNAI					
*month 3*					
	Patients - median (N)	Family members-median (N)	Test Statistic* - U	Effect size (2)	p**
Trust mental healthcare system	5 (1)	5 (1)	8295	0	0.754
Trust doctors	4.7 (0.8)	5.0 (0.7)	10242	0.028	0.004
Trust case managers	4.6 (0.7)	5.0 (0.7)	9665		0.054
*month 12*										
	Patients - median (IQR)	Family members-median (IQR)	Test Statistic* - U	Effect size (2)	p**
Trust mental healthcare system	5 (1)	5 (0)	11345	0.08	<0.001
Trust doctors	4.6 (0.7)	5.0 (0.5)	10592	0.027	0.004
Trust case managers	4.7 (0.7)	5.0 (0.5)	10664	0.03	0.003
*month 24*										
	Patients - median (IQR)	Family members-median (IQR)	Test Statistic* - U	Effect size (2)	p**
Trust mental healthcare system	4 (1)	5 (0)	14239	0.086	<0.001
Trust doctors	4.9 (0.5)	5 (0.4)	12699	0.03	0.003
Trust case managers	4.7 (0.7)	5 (0.4)	13236	0.04	<0.001

* Mann-Whitney Test;

** Correlation is significant at the 0.05 level (2-tailed).

## Data Availability

Ethics approval for this study and associated consent procedures do not allow us to publish datasets in a publicly accessible repository. However, the data that support the findings of this study are available from the corresponding author, S.N.I., upon reasonable request.
